# Comparison of Blended Learning With Traditional Dermatology Learning for Medical Students: Prospective Evaluation Study

**DOI:** 10.2196/49616

**Published:** 2024-02-01

**Authors:** Cristiana Silveira Silva, Cidia Vasconcellos, Murilo Barreto Souza, Juliana Dumet Fernandes, Vitoria Regina Pedreira de Almeida Rego

**Affiliations:** 1 Department of Dermatology, Federal University of Bahia Salvador, BA Brazil; 2 Department of Dermatology, University of São Paulo São Paulo Brazil; 3 Department of Ophthalmology, Federal University of Bahia Salvador Brazil

**Keywords:** dermatology, distance education, distance learning, e-learning, medical education, undergraduate medical education

## Abstract

**Background:**

Novel internet-based applications and associated technologies have influenced all aspects of society, ranging from commerce and business to entertainment and health care, and education is no exception. In this context, this study was designed to evaluate the impact of a dermatology e-learning program on the academic performance of medical students in dermatology.

**Objective:**

The aim of this study is to develop a dermatology blended-learning course for undergraduate medical students, evaluate the knowledge gained by students exposed to this course, and compare the results to those of traditional teaching methods.

**Methods:**

In this prospective study, we evaluated the performance of fourth-semester medical students at the Federal University of Bahia, Brazil. Students who had been in their second year of the medical course in 2019 were considered the control group, while students in their second year in 2020 were considered the blended or hybrid group. The first group attended traditional classes, using printed material (books and handouts), while the second group used our web-based course and e-book as a supplement in a hybrid web-plus-traditional fashion. Neither participants nor evaluators were blinded. The students in both groups were subjected to the same pre- and postcourse face-to-face, multiple-choice, paper-based evaluations, and we compared their performances. The content of the classes was the same for both groups. All didactic activities were developed by a team of certified dermatologists and professors from the university.

**Results:**

A total of 129 students were selected and divided into 2 groups: the control group (n=57) and the hybrid group (n=72). The precourse tests did not indicate any difference between the control group (mean score 2.74, SD 1.25) and the hybrid group (mean score 3.2, SD 1.22 SD; *P*>.05). The hybrid group had better final-term grades (mean 8.18, SD 1.26) than the traditional group (mean 7.11, SD 1.04). This difference was statistically significant (*P*<.05).

**Conclusions:**

This study explores pedagogical possibilities in the field of dermatology teaching for medical school students. The results suggest that the performance of undergraduate students who attended the course with additional e-learning material was superior when compared to the performance of those who participated in the traditional course alone.

## Introduction

During medical school, dermatological teaching in various countries, including the United Kingdom, is usually restricted [1,2]. Students are exposed to the topic as part of short-term internships or as an optional discipline [1,2]. In some institutions, dermatology disciplines are not even offered [1,3]. Published surveys in different countries have demonstrated that the amount of time devoted to dermatology in the medical student curriculum represents only 0.24%-0.3% of the 4 years of study [1]. In the last few years, even after curricular reformulation, the time devoted to teaching dermatology has decreased or remained the same [4-6]. McCleskey et al [4] found that only 10% of medical schools require a clinical dermatology rotation and that 93% of institutions offer dermatology as an elective rotation, usually a 4-week clerkship.

The current available time for dermatology training in medical schools worldwide is insufficient to learn about the various cutaneous diseases that students are likely to encounter in their future medical practice [5,7]. In view of this reality, the use of technologies that are able to optimize learning in dermatology has a great impact.

A meta-analysis evaluating the efficacy of educational interventions that improve diagnostic dermatological skills found that a blended curriculum that integrates multiple modalities of clinical dermatology teaching may be the most effective approach to meeting learning objectives [3]. The results observed by Lujan and DiCarlo [8] showed that first-year medical students learn through a variety of learning styles, with only 36.1% preferring a single way of acquiring new information. In recent years, we have noticed a growing interest among researchers in using new technologies to improve medical education [9-11]. The use of e-textbooks, podcasts, anatomical models, and virtual and interactive 3D computer models has positively impacted the educational experience of medical students [9]. Students exposed to interactive technology tools during their learning period demonstrate significant improvement on their performance tests [11].

Despite existing evidence that web-based teaching tools associated with interconnected content, when carefully selected, can assist the learning process, conventional teaching methods are still mainstream in medical teaching [12-14]. Teaching is mainly conducted in the form of hall lectures and laboratory sessions [1,12]. Despite large investments, there is a lack of sufficient evidence to support the effectiveness of digital interventions in the education of health professionals [15].

This study explores some pedagogical possibilities in the field of dermatology teaching for medical school students. It evaluates the use of web-based tools and an e-book developed specifically for this purpose, explores their impact on medical students’ learning, and compares this form of learning with traditional learning.

## Methods

### Overview

In this paper, we analyze the impact of web-based teaching tools on the performance of medical students at the Federal University of Bahia (UFBA), Brazil, and compare the results with those of traditional learning. Hence, we conducted a prospective study including medical students with computer literacy in the fourth semester at UFBA who were studying dermatology between June 2019 and June 2020. All the content was set in and developed in Brazil.

Students who had been in their second year of the medical course in 2019 were considered the control group, while students in their second year in 2020 were considered the blended group. The students were randomly allocated into the control or blended groups, and neither participants nor evaluators were blinded. All didactic activities were developed by a team of certified dermatologists and professors from UFBA.

All students participated in face-to-face activities. The classes included patient care in a general dermatology outpatient clinic. During the care, dermatological physical examination findings were emphasized, and the students were instructed to identify patients’ skin lesions and describe them according to the teaching material provided.

In the control group (traditional learning), after treating patients, students participated in an expository class structured into eight modules: (1) semiology, (2) leprosy, (3) syphilis, (4) atopic dermatitis, (5) skin virosis, (6) pyodermitis, (7) superficial mycosis, and (8) skin cancer. Doubts about the modules were clarified on this occasion.

The hybrid activities were composed of 5 distinct stages. In the first stage, we made a photographic record of patients who had dermatological lesions during a medical consultation held at the dermatology outpatient clinic at UFBA. In the second stage, we wrote a book (*Manual of Dermatology* [16]) using the cases cataloged in the first stage. During the third stage, we planned and prepared the web-based course according to predetermined modules. For each module, a video lesson was made available, lasting an average of 30 minutes, and the Camtasia (TechSmith) program was used for this activity. The video lessons were formatted and published on the Moodle (Moodle HQ) platform. The fourth stage comprised an e-learning module that included an 8-week course administered simultaneously with face-to-face classes.

The students in both groups were subjected to the same pre- and postcourse face-to-face evaluations, and their performances were compared. A total of 40 multiple-choice questions were written in accordance with the recommendations of the National Council of Medical Examiners to compose the pre- and postcourse exams [17]. To evaluate the validity of the content, 2 independent dermatologists examined all questions. The subject of the tests was chosen in accordance with the British Association of Dermatologists’ Undergraduate Curriculum [18].

Students in the control and hybrid groups received identical evidence-based content, and the courses had the same 8-week duration. The e-learning course was developed using the open-source Moodle learning management system.

Students logged in using individual usernames and passwords. A new text, video, and web-based discussion forum that addressed the same content as the face-to-face classes was available each week in an asynchronous mode. The students received weekly email notifications that a new class was available. In addition to face-to-face communication, students in the hybrid group could receive feedback on the discussion boards or by sending direct messages to the tutor. A 40-question multiple-choice test was given to all students in both groups before and after the courses, with scores ranging from 0 to 10. In the fifth stage, we analyzed the results using Stata (version 13.1; StataCorp) and Microsoft Excel (version 2007; Microsoft Corporation). Initially, the studied variables were evaluated in a descriptive manner, with the data presented as mean (SD) or median (IQR). The Shapiro-Wilk test was used to test normality. Pre- and postcourse scores obtained for each group were compared (intragroup comparisons). The results obtained in the pre- and postcourse tests were also compared between the control and hybrid groups (intergroup comparisons). According to the normality test, a 2-tailed paired *t* test, or Wilcoxon signed rank test, was applied for intragroup comparisons and a 2-tailed *t* test, or Mann-Whitney *U* test, for intergroup comparisons. The internal consistency of the pre- and postcourse assessments was evaluated using Cronbach α coefficients.

### Ethical Considerations

This study was approved by the Ethics Committee of the UFBA (1688.502) and conducted in accordance with the Declaration of Helsinki. Informed consent was obtained from all students. This work was not supported by any funding or external support, and no artificial intelligence resources were used.

## Results

A total of 129 students were included in this study. The average age was 23 (SD 1.3) years, and 62 students were male. No significant differences were found between the 2 groups in relation to sex and age. The control group (n=57) used traditional classroom paper-based tool activities, while the hybrid group (n=72) used our e-learning course and e-book made specifically for this course in a hybrid web-plus-traditional fashion. Demographic data per group are presented in Table 1. All participants completed the study. The mean pretest score for the control group was 2.74 (SD 1.25) and for the hybrid group, the mean pretest score was 3.2 (SD 1.22; *P*>.05). The final posttest mean score was 7.11 (SD 1.04) for the control group and 8.18 (SD 1.26) for the hybrid group. The intragroup comparisons of pre- and postcourse scores obtained for each group were statistically significant (*P*<.05).

**Table 1:** Demographic data.

Course	Male, n (%)	Female, n (%)	Age (years), mean (SD)
Hybrid group (n=57)	27 (47)	30 (53)	24 (1.2)
Control group (n=72)	35 (49)	37 (51)	22 (1.3)

**Figure 1:**
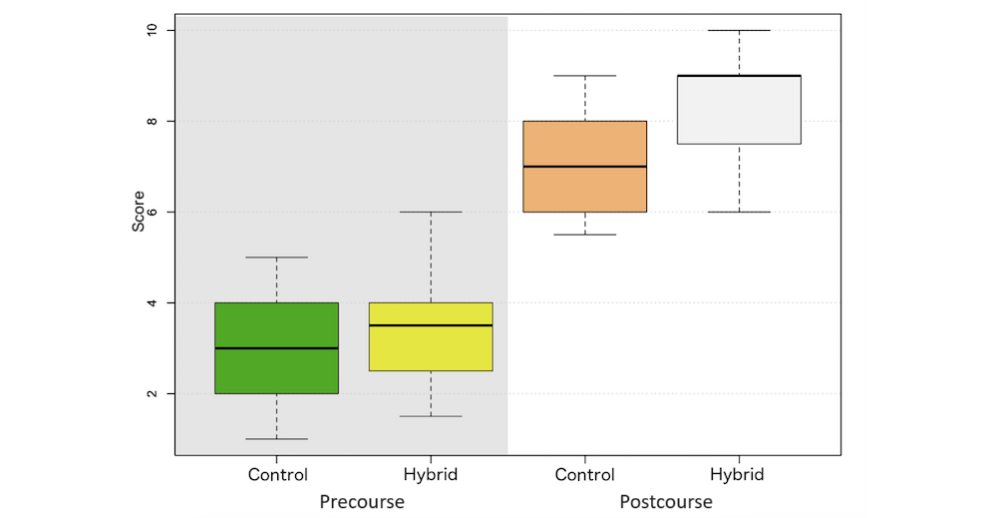
Boxplot with median (IQR) and range (minimum-maximum) illustrating the pre- and postcourse scores in both the conventional and hybrid groups.

Intergroup comparisons of the pretest scores demonstrated that there was no significant difference between the control and hybrid groups (*P*>.05), indicating that the baseline knowledge for each group was comparable (Figure 1). The results indicated increased scores in the hybrid group, implying the hybrid delivery method outperformed the traditional approach. A statistically significant difference in the postcourse scores between the 2 groups was achieved.

## Discussion

### Overview

The results of this study expand earlier findings on how hybrid learning can enhance learning outcomes in medical education. Moreover, this model was found to increase the effectiveness of teaching and learning methods in improving knowledge acquisition, which is consistent with the results of several other studies.

Although dermatology is essentially a visual specialty with great potential to benefit from today’s digital technologies, the conventional way of teaching still prevails [13,19]. At our university, medical education follows a traditional lecture-based curriculum, and this was the first time that digital technology was used. Nearly all aspects of web-based education were new and had to be understood [20]. We know that e-learning offers medical schools powerful and flexible learning resources [21] and presents several advantages, including (1) increased monitoring of student progress in a simpler and more accurate manner [8]; (2) the possibility of watching classes several times at more convenient times and places [8]; and (3) allowance for more than one way of student-teacher communication by means of emails, chats, and online discussion forums [22,23]. This last point is an advantage from the students’ point of view—although it may come at the expense of teachers’ time, as it has the potential to consume more of their time when compared with classroom teaching alone (where teachers are only available during class time or office hours) [13,23]. Web-based teaching also allows medical training to continue even in difficult situations (eg, the COVID-19 pandemic), and the greatest benefit is the flexibility offered by teaching platforms [24].

While e-teaching has real advantages, as discussed above, it also comes with some drawbacks. For example, this method does not support direct contact with the teacher or the patient, which may limit the observation of certain diseases and their diagnosis. Moreover, the method is dependent on the availability of electronic devices with adequate internet access [9] and needs a highly educated, motivated, and expert core team of teachers [18]. Silva et al [13] found the same challenges in web-based courses and described a significant technical difficulty in producing educational material for distance learning. The authors also highlighted great difficulty in facilitating students’ engagement with each other and in assessing the acquisition of practical skills in dermatology.

The provision of high-quality e-learning is highly labor-intensive. Like Fordis et al [25], we realized that the work spent on making web-based activities was more challenging than face-to-face teaching, especially when considering the design, organization, delivery, and engagement of participants in the discussion. A combination of both methods appears to be the best strategy [22,23,26]. In this study, these limitations were circumvented, as face-to-face activities were performed in both groups, and the students were given face-to-face contact time with both the teacher and patients seen at the clinic. Although some individuals report visual discomfort and others prefer reading a print book, both this study and the literature support the use of e-book technology in modern medical curriculum as an adjunct to traditional methods [9].

In this study, the full e-book content was available for download and could be accessed at any time, regardless of internet access. One of the main concerns about the switch to web-based lectures is the possible difficulty of lengthy readings on a screen and students’ ability to focus on reading. There is a great advantage to reading on the screen of an electronic device, as it allows for an increase in the font and size of the image, which facilitates assimilation of the content and helps individuals with reading difficulties [9]. Singer and Alexander’s [27] results indicated a clear preference of their participants for digital texts, as they generally achieved a better understanding when reading digitally [27]. However, the higher degree of satisfaction on the part of the student was not necessarily compatible with the results obtained in subsequent evaluations [24]. The vast majority approved the use of new technologies for dispensing the dermatological subject, and there were no complaints about this approach in this study.

It is currently believed that although reading on a computer screen may be more superficial and occasionally less accurate, it is the quality of the image presented to the reader that is crucial for the best use of the reading book [9]. Although there was a greater gain in knowledge in the group exposed to the distance e-learning associated with our e-book, some considerations must be made regarding the limitations and difficulties found in this study.

First, the evaluation was conducted in just one institution; ideally, more studies in multiple teaching centers with different realities from ours would be necessary for e-learning to consolidate itself as an effective form of education. Second, since the e-book was written especially for medical students, it is possible that its content made knowledge more accessible and didactic to the hybrid group, whereas the traditional group had to use renowned but conventional dermatology books. Third, we must mention the fact that the students in the traditional group spent 1 hour less per week on practical activities, totaling a reduction of 8 hours from their on-site internship due to the period spent in the in-person theoretical classes. Thus, students in the hybrid group received 8 hours more exposure to practical classes since the theoretical classes were attended at home. This difference may have favored the hybrid group in relation to obtaining better grades. In addition to better grades, increasing the time exposed to the discipline is one of the goals we strive to achieve in dermatological education.

The participants in the 2 groups had different admission years and were asked to maintain the contents and evaluations of the class confidential; however, we did not check for contamination between the 2 groups.

The field of education is destined to evolve. The professor is not the ultimate gatekeeper of definite knowledge; they also learn from students and need to incorporate feedback into the curriculum [28]. Despite this, the highest-quality clinical dermatology education will always require guided clinical exposure and feedback [18]. Innovative technologies cannot replace the need for enthusiastic and knowledgeable clinical teachers [1,28].

### Conclusion

This study explores pedagogical possibilities in the field of dermatology teaching for medical school students. The results suggest that the performance of undergraduate students who attended the course with additional e-learning material was superior to the performance of those who participated in the traditional course alone.

## Abbreviations

UFBA: Federal University of Bahia

## Data Availability

The data sets generated during and/or analyzed during this study are not publicly available due to privacy or ethical restrictions but are available from the corresponding author on reasonable request.
